# The age of the obesity onset is a very important factor for the development of metabolic complications and cardiovascular risk in children and adolescents with severe obesity

**DOI:** 10.1007/s00431-024-05636-x

**Published:** 2024-06-15

**Authors:** Ewa Szczudlik, Anna Stępniewska, Mirosław Bik-Multanowski, Stephanie Brandt-Heunemann, Bertram Flehmig, Ewa Małecka-Tendera, Artur Mazur, Elżbieta Petriczko, Michael B. Ranke, Martin Wabitsch, Agnieszka Zachurzok, Małgorzata Wójcik

**Affiliations:** 1 Department of Pediatric and Adolescent Endocrinology, Pediatric Institute, Jagiellonian University Medical College, Cracow, Poland; 2 Department of Medical Genetics, Faculty of Medicine, Jagiellonian University Medical College, Cracow, Poland; 3 Children’s Hospital, University of Tübingen, Tübingen, Germany; 4 Center for Rare Endocrine Diseases, Division of Pediatric Endocrinology and Diabetes, Department of Pediatrics and Adolescent Medicine, Ulm, Germany; 5 Mediagnost GmbH, Reutlingen, Germany; 6 Department of Pediatrics and Pediatric Endocrinology, Medical University of Silesia, School of Medicine in Katowice, Katowice, Poland; 7 Department of Pediatrics, Pediatric Endocrinology and Diabetes, Medical Faculty, University of Rzeszów, Rzeszów, Poland; 8 Department of Pediatrics, Endocrinology, Diabetology, Metabolic Disorders and Cardiology of Developmental Age, Pomeranian Medical University, Szczecin, Poland; 9 Department of Pediatrics, Faculty of Medical Sciences in Zabrze, Medical University of Silesia, Zabrze, Poland; 10 Institute of Human Genetics, University Hospital, LMU, Munich, Germany

**Keywords:** Severe obesity, High blood pressure, Metabolic complications, Children, Adolescents

## Abstract

Severe obesity defined as BMI value corresponding to an adult > 40 kg/m^2^ affects 1–5% of children and adolescents in Europe. The purpose of this study was to assess the occurrence of cardiovascular risk factors in children and adolescents with severe obesity. The analysis included 140 patients (75 female) at the mean age of 14 ± 2.1 SD (range 10–18) years (all recruited in 4 regional reference centers in Poland). Severe obesity was defined as BMI > 35 kg/m^2^ (children 6–14 years), and BMI > 40 kg/m^2^ (> 14 years). Fasting plasma samples have been obtained in all patients, and OGTT was performed in all patients. The metabolic risk factors were defined as high blood pressure (BP > 90 percentile for height, age, and sex), HDL cholesterol < 1.03 mmol/L, TG ≥ 1.7 mmol/L, and hyperglycemic state (fasting blood glucose > 5.6 mmol/L, or blood glucose 120′ after oral glucose load > 7.8 mmol/L). Additionally, the *MetS z-score* was calculated using Metabolic Syndrome Severity Calculator. One hundred twenty-four (89%) participants presented with high BP, 117 (84%) with abnormal lipid profile, and 26 with the hyperglycemic. Only 12 (9%) were free of metabolic complications. More than 60% of patients had more than one cardiovascular risk factor. The high BP was significantly associated with the severity of obesity (*F* = 9.9, *p* = 0.002). Patients with at least one metabolic complication presented with significantly younger age of the onset of obesity (the mean age of the patients with no overt obesity complications was 10 years, while the mean age of those who presented at least one was 4.7 ± 3.5 SD years (*p* = 0.002)). A significant positive association between in the value of the *Mets BMI z-score* with age was observed (*R* = 0.2, *p* < 0.05). There were no differences between girls and boys regarding *Mets BMI z-score* (1.7 ± 0.8 vs 1.7 ± 0.7, *p* = 0.8).

*Conclusions*: The most common metabolic risk factor in children and adolescents with severe obesity was high BP. The most important factor determining presence of obesity complications, and thus the total metabolic risk, seems to be younger (< 5 years) age of onset of obesity.

**Table:** 

**What is Known?** • *It is estimated that 1-5% of children and adolescents in Europe suffer from severe obesity corresponding to an adult BMI > 40 kg/m2, and it is the fastest growing subcategory of childhood obesity.* • *Children with severe obesity face substantial health risk that may persist into adulthood, encompassing chronic conditions, psychological disorders and premature mortality.*
**What is new:** • * The most common complication is high BP that is significantly associated with the severity of obesity (BMI z-score), contrary to dyslipidemia and hyperglycemic state, which do not depend on BMI z-score value.* • *The most important factor determining presence of obesity complications, and thus the total metabolic risk, seems to be younger (< 5 years) age of onset of obesity.*

**What is Known?**

• *It is estimated that 1-5% of children and adolescents in Europe suffer from severe obesity corresponding to an adult BMI > 40 kg/m2, and it is the fastest growing subcategory of childhood obesity.*

• *Children with severe obesity face substantial health risk that may persist into adulthood, encompassing chronic conditions, psychological disorders and premature mortality.*

**What is new:**

• * The most common complication is high BP that is significantly associated with the severity of obesity (BMI z-score), contrary to dyslipidemia and hyperglycemic state, which do not depend on BMI z-score value.*

• *The most important factor determining presence of obesity complications, and thus the total metabolic risk, seems to be younger (< 5 years) age of onset of obesity.*

## Introduction

The term “morbid obesity” was introduced in mid-sixties of the twentieth century in order to justify insurance reimbursement for the cost of intestinal bypass surgery for weight loss in adult people with a BMI over 40 kg/m^2^ [1]. It defines severe, life-threatening excessive body weight. The terminology related to obesity is continually changing, and to respect for individuals dealing with this condition, it is now recommended rather to use “severe” instead of “morbid obesity” [2]. According to T.J. Cole and T. Lobstein, severe obesity in children can be defined as having a BMI of at least 35 kg m^2^, which can be also expressed as the 99.8th percentile at the age of 18 years [3]. Kelly et al. have defined severe obesity as BMI equal or above 99th percentile, BMI ≥ 120% of the 95th percentile, and as an absolute BMI ≥ 40 kg/m^2^ [2]. It is estimated that 1–5% of children and adolescents in Europe suffer from severe obesity corresponding to an adult BMI > 40 kg/m^2^. In the United States, the prevalence is even higher reaching 4–6% of the general pediatric population [4, 5]. What is particularly significant and worrying is the fastest growing subcategory of childhood obesity [2, 6]. Such a huge persistent excess of fatty tissue leads to the development of complications that increase the risk of heart and blood vessel diseases. Individuals with severe obesity face substantial health risk that may persist into adulthood, encompassing chronic conditions, psychological disorders and premature mortality [7–10]. The recent study revealed that adolescents with severe obesity face a greater than fourfold higher risk of a cardiovascular events within 30 years compared to normal-weight peers [11]. Nevertheless, typical, overt cardiovascular complications in children are much less common than in adults, despite similar BMI values. These risk factors with a common etiology may form clusters, and their negative effects may not only add up, but intensify. Moreover, each one disorder is not a separate disease, but a signal and an overt manifestation of impaired metabolism. The specific signs and symptoms are usually caused by a common underlying pathology, and their combination confers a risk that is different from the sum of the parts [12]. Therefore, comprehensive diagnostic and treatment, rather than activities aimed at improving individual metabolic parameters, is important.

The study aimed to assess the occurrence of cardiovascular risk factors in children and adolescents with severe obesity, to estimate the frequency of their co-occurrence and formation of clusters. Moreover, an attempt to assess the risk factors leading to the occurrence of single and grouped metabolic complications of obesity in children and adolescents with severe obesity.

## Material and methods

Patients included in the analysis were participants of a multicenter project conducted in four specialized medical centers in Poland (Szczecin, Cracow, Zabrze, Rzeszow). This is a prospective multi-center clinical study performed with the sample size targeted is 500 patients aged 1–18 years, with severe obesity of an early origin, hyperphagia, and food-seeking behaviors.

This paper describes preliminary, partial results of the project [13].

### Patients

The inclusion criteria were as follows: severe obesity defined as BMI > 35 kg/m^2^ (children in the age 6–14 years) and BMI > 40 kg/m^2^ (in older), written informed consent of the patient’s parent/guardian, and patient above the age of 13 years to participate in the study. The group was ethnically homogeneous.

### Methods

Anthropometric assessment was performed in all patients (body weight, height, waist circumference measurement, and body composition analysis using the bioelectrical impedance method). Body weight was measured to the nearest 0.1 kg on a certified medical scale, body height was measured to the nearest 0.1 cm on Harpenden stadiometer, waist circumference was measured at the level of midpoint between the lowest rib and iliac crest, and hip circumference was measured at the level of the greatest convexity of the buttocks on the back and with cardboard applied tangentially to the greatest convexity of the abdomen on the front by measuring tape to the nearest 0.5 cm. Bioimpedance measurements were conducted using TANITA MC-580 M S MDD, TANITA MC-780MA-N, and TANITA MC-780 P MA devices. The office blood pressure (BP) was assessed with certified, calibrated oscillometer devices. A cuff size was appropriate to the arm size, while a participant was in a sitting position following a 15-min rest period before the examination. Results were interpreted according to European Society of Hypertension reference values [14]. High BP was defined as values > 90th percentile for sex, age and height [14].

Biochemical markers of cardiovascular risk were measured in each patient in a single fasting blood sample collected in the morning. Glucose, triglyceride (TG), and HDL cholesterol levels were analyzed. In order to more precisely analyze glucose metabolism disorders, patients underwent an oral glucose tolerance test, with the assessment of blood glucose concentration 120 min later after glucose load (75 g). The lipids cut-off values were based on the definition of metabolic syndrome in children and adolescents by IDF consensus report: TG ≥ 1.7 mmol/L and HDL < 1.03 mmol/L [15]. The diagnostic criteria for IFG was a fasting blood glucose of 100–125 mg/dL (5.6–6.9 mmol/L), IGT was defined as a 2-h plasma glucose level between 140 and 199 mg/dl (7.8–11.0 mmol/L) and DM as a fasting glucose level of 126 mg/dL (7.0 mmol/L) or higher (two abnormal readings required) or a two-hour plasma glucose level of more than 200 mg/dL (11.1 mmol/L) (one abnormal reading required) [16]. The *MetS BMI z-score* was calculated using Metabolic Syndrome Severity Calculator [17, 18].

### Ethics approval statement

The study was conducted according to the guidelines of the Declaration of Helsinki “Ethical Principles for Medical Research in Humans” (9 July 2018). The study was approved by the local ethics committee (IRB)-Bioethics Committee of the Jagiellonian University (No. PCN/CBN/0022/KB1/137/I/21/22, KBETUJ 1072.6120.69.2022, KB-006/12/2022).

## Results

**Fig. 1:**
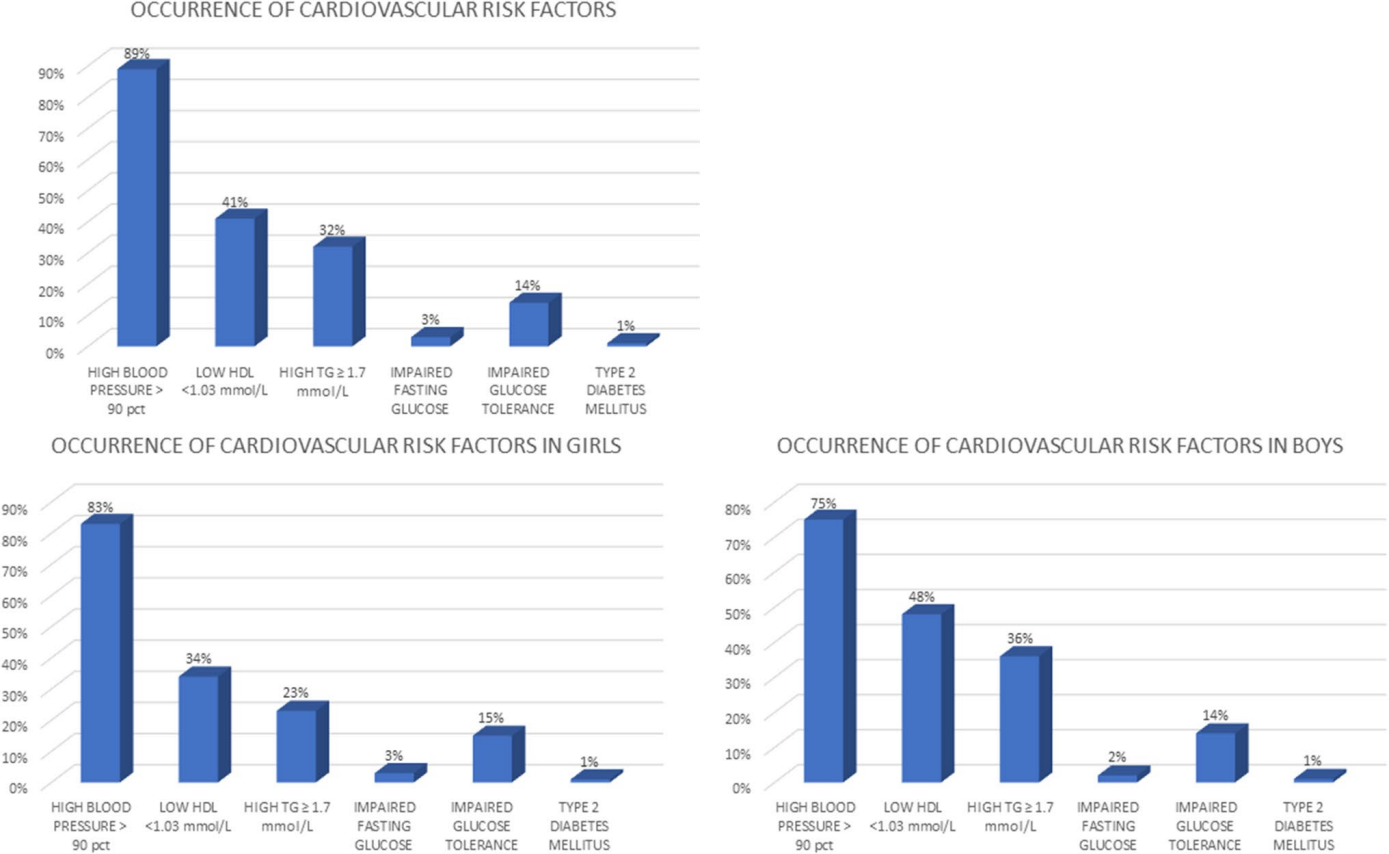
The occurrence of metabolic syndrome risk factors in the whole study group (*n* = 140, mean BMI *z-score* 3.7) and separately in girls and boys. No significant differences were found in the frequency of disorders depending on sex

**Fig. 2:**
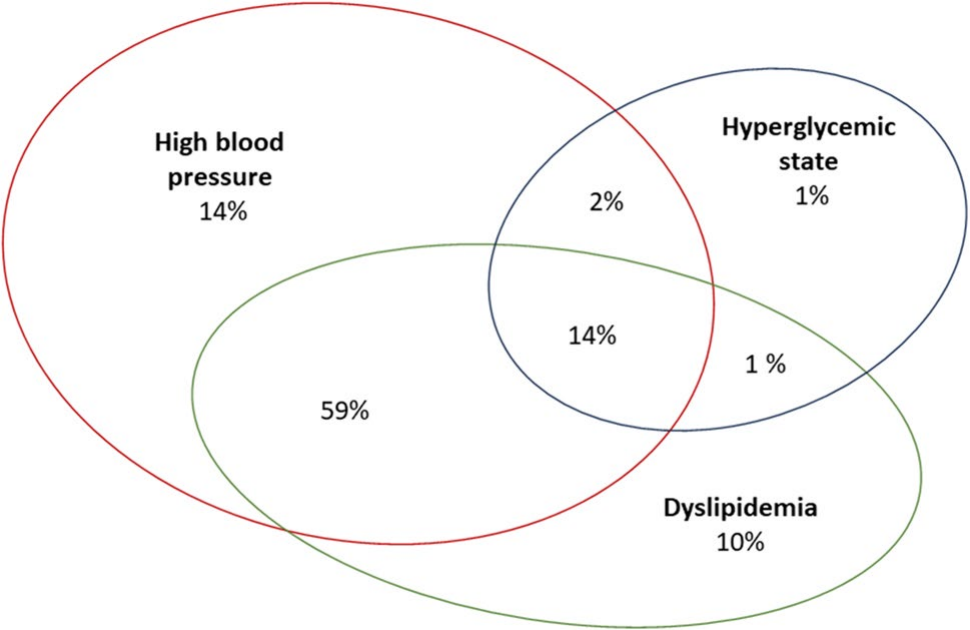
Clusters of the cardiovascular risk factors in the study group (*n* = 140, BMI *z-score* 3.7 ± SD)

**Fig. 3:**
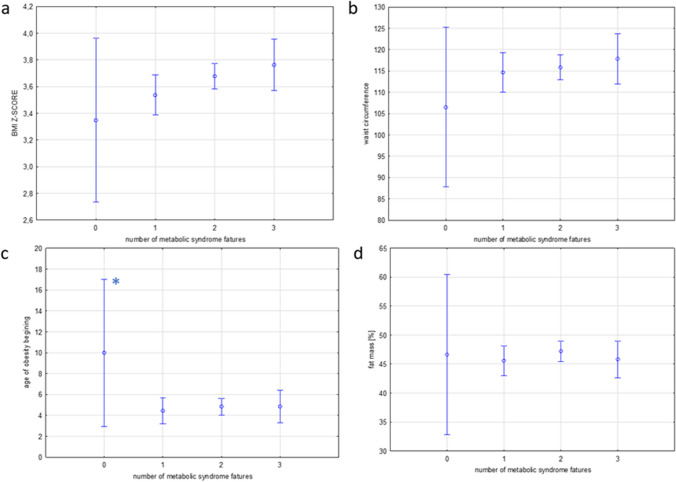
Differences in the mean BMI *z-score* (**a**), waist circumference (**b**), age of onset of obesity (**c**), and fat mass [%] (**d**) in dependency on the number of cardiovascular risk factors. *Statistically significant differences

The analysis included 140 patients (75 female) with severe obesity at the mean age of 14.0 ± 2.0 SD (range 10–18) years at time of the study. The mean BMI *z-score* in the study group was 3.7 ± 0.4 SD. Female patients presented with significantly lower BMI *z-score* (3.5 vs. 3.8, *p* < 0.001), but higher fat mass percent (48.3 ± vs. 44.6, *p* < 0.001) compared to male. They had also lower mean fasting glucose levels (4.8 vs. 5.0 mmol/L, *p* < 0.008) and higher mean HDL cholesterol levels (43.7 vs. 40.1 mg/dL, *p* = 0.01) compared to male patients (Table 1.). The present study revealed that the most common disorder in adolescents with severe obesity was high BP (diagnosed in 124 [89%] of participants) (Fig. 1). One hundred seventeen (84%) presented with abnormal lipid profile (58 [41%] with isolated low HDL, 44 [31%] isolated high TG, and 62 [44%] with both disorders), 26 [18%] with the hyperglycemic state (3 (2%) impaired fasting glucose, 20 (14%) impaired glucose tolerance, and 2 (1%) diabetes mellitus). No significant differences were found in the frequency of disorders depending on sex. Only 12 (9%) were free of the overt metabolic syndrome features (Fig. 1). A single metabolic complication was noticed in 33 (25%) of participants, two were present in 85 (62%), and three (high BP, hyperglycemic state, and dyslipidemia) were in 10 (14%) participants (Fig. 2). The most common combination was high BP and dyslipidemia (59%). High BP and disorders of glucose metabolism were present in 2% of patients and lipid disorders and glucose metabolism disorders in 1%. All three metabolism disorders were present in 14% of patients. Patients with the co-occurrence of 2 or more disorders were characterized by a younger age at the onset of obesity compared to patients with one or no complication (Fig. 3c). The mean age of the patients with no overt obesity complications was 10 ± 2.8 SD years, while the mean age of those who presented with 1.2 or 3 was 4.4 ± 2.0SD, 4.8 ± 2.1 SD, and 4.8 ± 1.8 SD, respectively. The difference was statistically significant when comparing the age of children without complications to those with at least one (*p* = 0.002). Differences in the age at which obesity occurred were not statistically significant when we compared groups with 1, 2, or 3 complications. The incidence of the number of cardiovascular risk factors increased with increasing BMI *z-score* but the relationship was not statistically significant (*p* = 0.2) (Fig. 3a). Similar effect was observed for waist circumference (mean 106.5 cm in those with no disorders and 114.6 cm, 115.8 cm, and 117.8 cm in those with 1, 2, and 3, respectively) (not shown). There was no significant difference regarding the fat mass % (46.6% in those with no disorders, and 46%, 47%, and 46% in those with 1, 2, or 3 respectively) (Fig. 3d). There was no association between birth weight and the number of metabolic complications of obesity.

**Table 1:** Characteristic of the study group

	Whole group (*N* = 140)	Girls (*N* = 75)	Boys (*N* = 65)	*p*-value
	(Mean ± SD)			
Age (years)	14.0 ± 2.0	14.1 ± 2.0	13.9 ± 2.0	0.56
Age of obesity onset (years)	4.8 ± 3.6	4.9 ± 3.6	4.6 ± 3.6	0.54
Birth weight (kg)	3.4 ± 0.5	3.4 ± 0.5	3.5 ± 0.5	0.21
BMI *z*-score	3.7 ± 0.4	3.5 ± 0.4	3.8 ± 0.5	0.000001*
Fat mass (%)	46.5 ± 8.2	48 ± 8.3	44.6 ± 7.3	0.004*
SBP (mmHg)	136 ± 13	133.7 ± 14.2	139 ± 12	0.2
DBP (mmHg)	81 ± 9.5	80.7 ± 8.7	81 ± 11	0.3
TG (mmol/L)	1.6 ± 0.9	1.5 ± 0.7	1.8 ± 1.1	0.06
HDL (mmol/L)	1.1 ± 0.2	1.1 ± 0.2	1.1 ± 0.2	0.01*
Fasting glucose (mmol/L)	4.9 ± 0.5	4.8 ± 0.4	5.0 ± 0.5	0.4
Gluose 120’ OGTT	5.0 ± 3.2	5.1 ± 3.3	4.9 ± 3.0	0.5

*Statistically significant differences

**Fig. 4:**
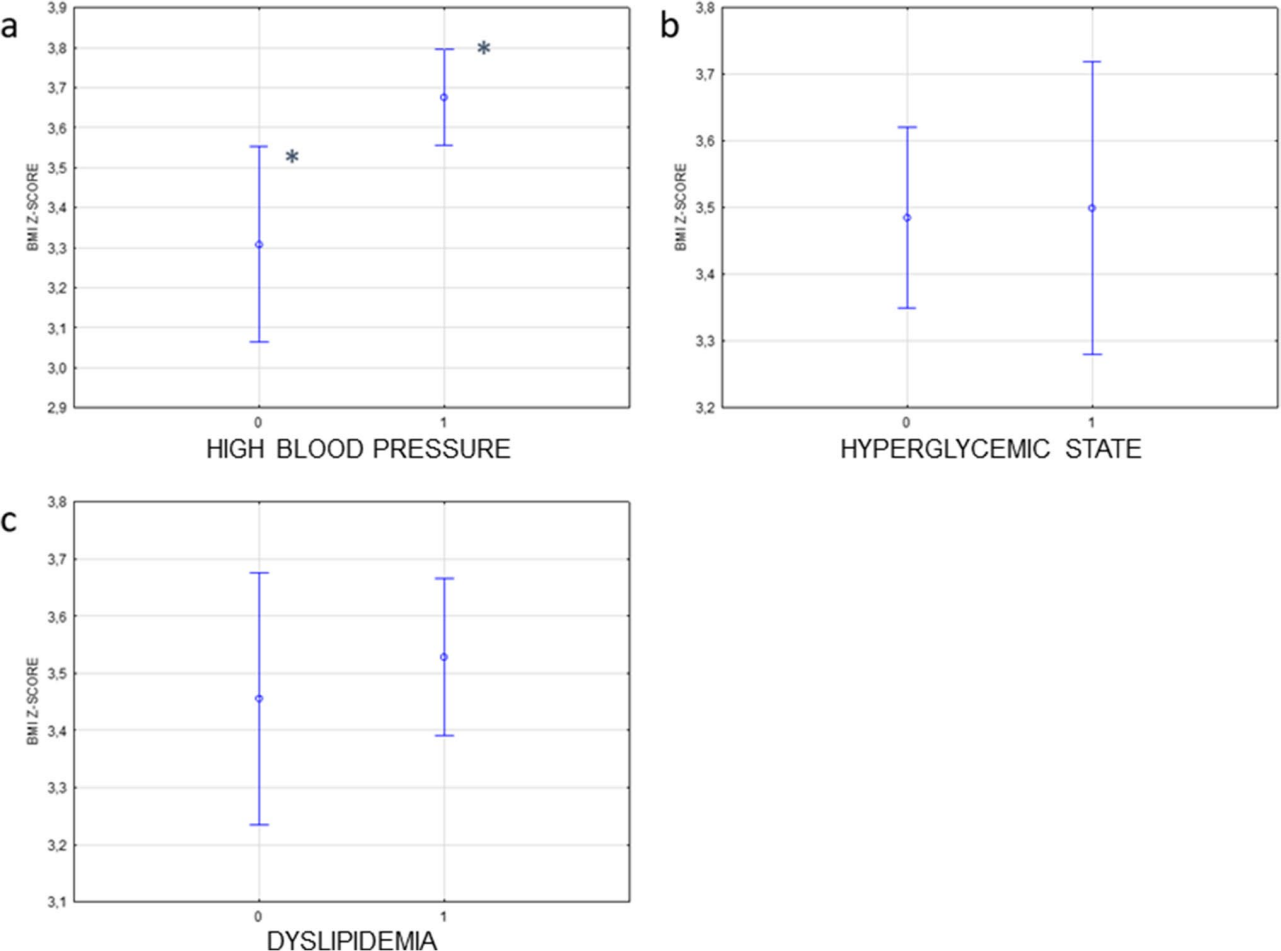
The relationship between the occurrence of cardiovascular risk factors and BMI *z-score*. Statistical significance was demonstrated for the relationship between BMI *z-score* and high blood pressure (**a**) (*F*(1, 132) = 9.9, *p* = 0.002). For hyperglycemic state [*F*(1, 132) = 0.02, *p* = 0.9] (**b**) and dyslipidemia (**c**) [*F*(1, 132) = 0.5, *p* = 0.5], the relationship with BMI *z-score* was not statistically significant. 0—no disorder, 1—disorder present. *Statistically significant differences

Multivariate regression analysis revealed that high BP was significantly associated with the severity of obesity (BMI *z-score*) [*F*(1, 132) = 9.9, *p* = 0.002]. For dyslipidemia [*F*(1, 132) = 0.5, *p* = 0.5, 95% CI] and for hyperglycemic state [*F*(1, 132) = 0.02, *p* = 0.9], the association with BMI *z-score* was not statistically significant (Fig. 4). Patients with high BP had significantly higher BMI *z-score* compared to patients with normal BP (3.7 ± vs. 3.3 ± , *p* = 0.001). The *Mets BMI z-score* increased with the age (*R* = 0.2, *p* < 0.05). There were no differences in *Mets BMI z-score* between girls and boys (1.7 ± 0.8 vs 1.7 ± 0.7, *p* = 0.8).

## Discussion

There is no doubt that obesity leads to the development of complications that constitute direct risk factors for heart and vascular diseases. The data from literature show, that in adult patients this cardiovascular risk is greater in people with severe obesity in comparison to moderate obesity [19]. In a cohort of Danish and Finnish subjects, each increase in BMI *z-score* at 7 years of age (equivalent to a 1.5 to 2.5 kg/m^2^ increment) was associated with a 5%-10% greater risk of coronary heart disease in adulthood [20]. In few studies conducted among teenagers with severe obesity, it was shown that they have a worse cardiometabolic risk profile including increased numbers of risk factors such as higher BP, dyslipidemia, diabetes mellitus, hyperglycemia, or hyperinsulinemia [9, 11–23]. It also appears that severely obese patients are predisposed to a greater number of cardiovascular risk factors, known as the metabolic syndrome [24]. The present study revealed that at least metabolic complication occurred in 91.5% of participants. It was only one disorder in 24%, two in 61%, and three in 7% of participants. This is a higher percentage than shown in a similar, but nationwide prospective surveillance study from the Netherlands, that showed at least one cardiovascular risk factor in 67% patients with severe obesity and two, three and more than three risk factors in 17%, 8%, and 2.5%, respectively. The most frequently reported cardiovascular risk factor was arterial hypertension [7]. A study from the USA reported results similar to our study: 84% of pediatric patients (age 5–17) with severe obesity had at least one cardiovascular risk factor [8]. In the study form the Netherlands, the most common complication of obesity in children and adolescents was arterial hypertension [7]. The incidence of high BP among children around the world is increasing in parallel with the increase in body weight [8, 24, 25]. This is also confirmed by the results of the present study, in which high BP was diagnosed in almost 90% of participants. Studies in adults have shown that the risk of hypertension increased according to the higher BMI [24], with the odds of hypertension as great as 4.8 among adults with class III obesity (BMI ≥ 40 kg/m^2^) when compared to adults with a normal BMI [26]. In the study by Sorof et al. the systolic BP increases progressively to BMI percentile [27]. In a cross-sectional retrospective US cohort, children with severe obesity and mild obesity had an odds ratio of 4 and 2 for hypertension, respectively, compared to children with normal weight [28]. Similarly in a nationwide cross-sectional Israeli cohort, odds ratios for hypertension ranged from 2.1 to 3.4 among girls and boys with severe obesity, and from 1.4 to 1.8 among those with mild obesity, compared to children with overweight [29]. In the most studies that assessed cardiometabolic risk factors according to BMI status, higher mean values of TG, and lower mean values for HDL were observed [21, 30–32]. Data from the literature shows that higher prevalence and increasing trends of abnormal values of HDL cholesterol, triglyceride (TG) levels, and systolic and diastolic BP were observed with increasing BMI categories [33–37]. The cross-sectional study by Norris AL who investigated 225 children adolescents with normal weight, overweight, obesity, and severe obesity revealed that values of BP, and the levels of insulin, and lipids worsened with the higher BMI [31]. In the present study, the presence of higher number of the complications increased parallel to BMI *z-score*; however, it was not statistically significant. The Bogalusa Heart Study established that schoolchildren with overweight were 2.4 to 7.1 times more likely to have elevated total cholesterol, LDL, and TG in comparison to thin peers [8]. The NHANES data indicate this pattern is highly prevalent, present in 42.9% of children with BMI > 95th% percentile. In observational, cross-sectional, retrospective study among Obese Children and Adolescents in Turkey the prevalence of dyslipidemia was 56.7%. A relatively low HDL-C together with a high TG was observed. From the participants, 644 (56.7%) cases met the study criteria for dyslipidemia, including 16% with high TC, 15% with high LDL-C, 36.8% with low HDL-C, and 25.9% with high TG. There was no difference in the frequency of dyslipidemia according to gender (*p* = 0.15). While dyslipidemia was seen in 62.3% of pubertal children, this was significantly less prevalent (49.4%) in prepubertal children (*p* < 0.001). Hypertriglyceridemia and low HDL-C were present at significantly higher rates in pubertal children (*p* < 0.001) [38]. Deeb et al. found that 55.3% of 216 children with obesity and overweight enrolled in the study had dyslipidemia [39]. Brzezinski et al. reported the incidence as approximately 69.9%. At least one of the lipid disorders occurred in 38.23% of girls and 40.51% of boys with overweight and obesity. The most common lipid disorders were decreased high-density lipoprotein cholesterol (HDL-C) levels (present in 20.55% of the girls and 23.79% of the boys) and elevated low-density lipoprotein cholesterol (LDL-C) (present in 15.31% of the girls and 14.25% of the boys) [40]. Nielsen et al. demonstrated the risk of developing lipid disorders is 2.8 times higher in obese children (BMI > 90th percentile) than in children with normal body weight [41]. The rarest disorder in the present study was hyperglycemia. According to the literature data in young adults, each 1-kg/m^2^ increase in BMI was associated with a 6% higher risk of developing type 2 diabetes before the age of 45 years [42]. In severely obese children and adolescents in cross-sectional studies, prediabetes was detected in 22–36% children and adolescents [43]. In the study by Propst et al. a retrospective electronic chart review was conducted on 1111 obese and morbidly obese children. Prediabetes was found in 19.5% of obese subjects and in 27.3% of morbidly obese ones. Moreover, 39.8% of obese children and 52.4% of morbidly obese ones had type 2 diabetes [44]. The present study is the first attempt to analyze the potential factors contributing to the development of metabolic complications in pediatric patients with severe obesity. There was an association between the age of the onset of obesity and development of cardiovascular risk. Interestingly, the presence and number of risk factors was not associated with fat mass, nor waist circumference. Contrary to adults in whom waist circumference is considered as an independent risk factor for metabolic syndrome, more important than BMI itself [19]. In the present study the incidence of more complications increased parallel to BMI *z-score* (mean BMI *z-score* 3.3 in those with no overt cardiovascular risk factors to 3.5, 3.7, and 3.8 in those with 1, 2, or 3 disorders), but the relationship was not statistically significant (*p* = 0.2).

### Limitations of the study

The main study limitation is the relatively modest size of the group. The inclusion criteria focused on children and adolescents with extreme obesity, which naturally impacts the number of patients. Moreover, this is a relatively underrepresented group in existing literature. An important factor to consider is that there was a marked disproportion between the number of children in the complication-free subgroup and those with 1, 2, or 3 disorders. Due to the frequent occurrence of disorders in children and adolescents with severe obesity, it would probably be difficult to gather a sufficiently large group free from cardiovascular risk factors. Another limitation pertains to the fact that biochemical and hormonal tests, as well as BIA measurements, were conducted in different laboratories using different equipment. Nonetheless, all gathered data were compared against center-specific standards.

## Conclusions

The most common cardiovascular risk factor in children and adolescents with severe obesity was high BP. Over 60% of patients had more than one cardiovascular risk factor. The most important factor determining presence of obesity complications, and thus the total metabolic cardiovascular risk, seems to be younger (< 5 years) age of onset of obesity.

## Data Availability

Data can be provided on demand, after a contact with corresponding author.

## Abbreviations

BMI: Body mass index
BP: Blood pressure
DM: Diabetes mellitus
HDL: High-density lipoprotein
LDL: Low-density lipoprotein
IFG: Impaired fasting glucose
IGT: Impaired glucose tolerance
OGTT: Oral glucose tolerance test
TG: Triglycerides

## Declarations

### Open access

For the purpose of Open Access, the authors have applied a CC-BY public copyright license to any Author Accepted Manuscript (AAM) version arising from this submission.
